# Spermatic Cord Lymphoma: A Case Report and Literature Review

**DOI:** 10.1155/2012/513707

**Published:** 2012-02-06

**Authors:** Satoru Taguchi, Sayuri Takahashi, Katsuyuki Iida, Takashi Mizutani, Kazumi Yamaguchi, Takashi Tominaga, Naoya Niwa, Mayumi Yoshimi, Tsuyoshi Takahashi, Yukio Homma

**Affiliations:** ^1^The Department of Urology, Mitsui Memorial Hospital, Tokyo 101-8643, Japan; ^2^The Department of Urology, Faculty of Medicine, The University of Tokyo, Tokyo 113-8655, Japan; ^3^The Department of Hematology, Mitsui Memorial Hospital, Tokyo 101-8643, Japan

## Abstract

Spermatic cord lymphoma is a rare lethal disease. It has a poor prognosis even in stage I or II disease when treated locally, therefore, multidisciplinary treatment for early stage is recommended. On the other hand, the treatment of choice for stage III or IV spermatic cord lymphoma remains to be determined. It is said that spermatic cord lymphoma is clinicopathologically similar to primary testicular lymphoma, therefore the treatment of spermatic cord lymphoma has often been determined by reference to the recommended treatment for primary testicular lymphoma. Here we report a new case of spermatic cord lymphoma, which was found in stage IV disease. We also review thirty-three cases which have been reported as spermatic cord lymphoma to date, and discuss treatment options.

## 1. Introduction

Spermatic cord lymphoma is a rare lethal disease. To our knowledge, searching both English and Japanese literature, only thirty-three cases including our case have been reported [1–8]. It has a poor prognosis even in stage I disease when treated locally, therefore, multidisciplinary treatment for early stage is recommended. Here we report a new case of spermatic cord lymphoma, which was found in stage IV disease. We also review literature and discuss treatment options.

## 2. Case Report

**Figure 1:**
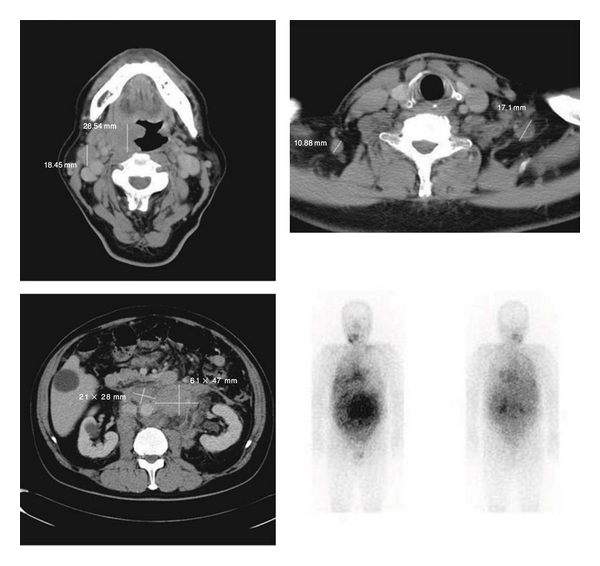
Computed tomographic scan (CT) showing the swelling of the right tonsil and the enlargement of right cervical, left supraclavian, and para-aortic lymph nodes. 67 Ga scintigraphy showing intense uptake in the same lesions which were detected in CT.

**Figure 2:**
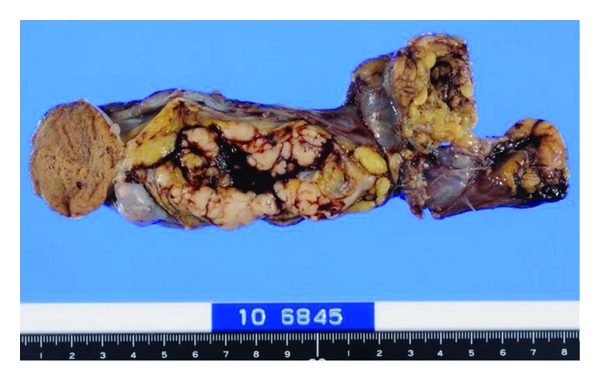
Gross appearance of the surgical specimen showing a white 67 mm tumor around the left spermatic cord.

**Figure 3:**
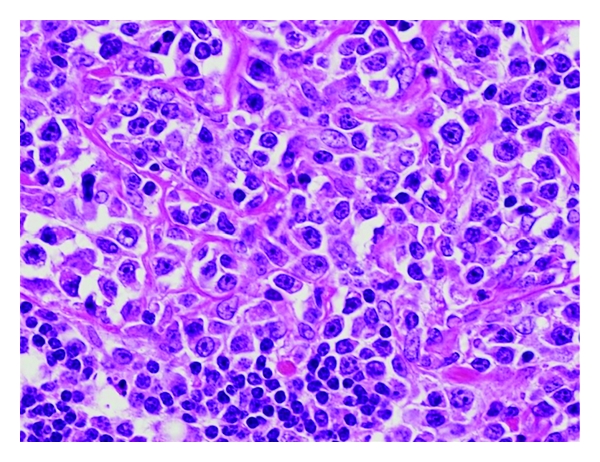
Microscopic features of the tumor consisting of proliferative large atypical lymphocytes.

In July 2010, a 62-year-old man visited an urologist with a complaint of a left intrascrotal mass and was placed under clinical observation without treatment. Two months later, a swelling of the right tonsil appeared, and he visited an otorhinolaryngologist. After a biopsy of the right tonsil, he was diagnosed with diffuse large B-cell lymphoma (DLBCL) and was admitted to our hospital. Physical examination showed that the right tonsil swelled up beyond the midline, right cervical and left supraclavian lymph nodes were palpable, and a 50-mm mass existed along the left spermatic cord. He also had night sweats. In the blood examination, high levels of serum soluble interleukin-2 receptor were detected (5290 U/mL). Ultrasonography showed a 50 mm tumorous lesion in maximum diameter in the left spermatic cord and a 5 mm mass in the right spermatic cord, respectively. Computed tomographic scan (CT) showed the swelling of the right tonsil and the enlargement of right cervical, left supraclavian, and para-aortic lymph nodes. 67 Ga scintigraphy showed intense uptake in the same lesions which were detected in CT, but no uptake in groins (Figure 1). Bone marrow aspiration showed a few large atypical cells that were positive for CD20 and negative for CD3, which suggests the invasion of lymphoma to the bone marrow. Based on these findings, he was clinically diagnosed as DLBCL at stage IVB (Ann Arbor Staging). In December 2010, he underwent left radical orchiectomy for confirming the diagnosis and mass reduction. Macroscopically, a white 67 mm tumor in maximum diameter existed around the left spermatic cord, but the testis and the epididymis were not involved (Figure 2). It consisted of proliferative large atypical lymphocytes (Figure 3). Immunohistochemistry revealed that the tumor cells were positive for CD20 and negative for CD3. Histopathological diagnosis was DLBCL of the left spermatic cord. As adjuvant therapy, he completed both 6 cycles of rituximab added to cyclophosphamide, doxorubicin, vincristine, and prednisone (R-CHOP) and 4 cycles of intrathecal methotrexate (IT-MTX) for central nervous system (CNS) prophylaxis. Then, he also underwent radiotherapy (RT) to the right testis for prophylaxis of contralateral testicular relapse. CT performed 4 months after the orchiectomy showed that all lesions either disappeared or reduced, and that there was no new lesions.

## 3. Discussion

Male gonadal involvement is relatively uncommon in malignant lymphoma. Of these lymphomas, testicular lymphoma is the most frequent, accounting about 1% of all nonHodgkin's lymphomas [9]. On the other hand, spermatic cord lymphoma is very rare, and to the best of our knowledge, only thirty-three cases including our own case have been reported (Table 1) [1–8]. The patients ranged in age from 20 to 89 years (mean age, 60-year-old). Middle-aged men (14 out of 33 cases) and older men (15 out of 33 cases) were mainly affected [10]. Most cases were stage I disease at the time of diagnosis (stage I, 21 cases; stage II, 6 cases; stage III, 2 cases; stage IV, 4 cases), nevertheless, they apparently have a poor prognosis (median survival, 12.2 months). Our case was already in stage IV at presentation. In general, it is difficult to detect the primary site when a patient with malignant lymphoma has multiple lesions. However, the first manifestation of our case was the spermatic cord tumor. Spermatic cord lymphoma has a tendency to spread or relapse towards para-aortic lymph nodes, while lymphomas derived from different primary lesions hardly metastasize to a spermatic cord in an early stage [5]. Therefore, we assumed the primary site in our case might be the spermatic cord. Histologically reviewing, intermediate-grade lymphomas (Working Formulation), centroblast or centroblastic-centrocytic lymphomas (Kiel classification), and DLBCL (WHO classification) were most frequently observed, except two cases which suffered from Burkitt's subtype [1, 4]. All cases that the immunophenotypical study was performed showed a B-cell phenotype.

It is said that spermatic cord lymphoma is clinicopathologically similar to primary testicular lymphoma (PTL). PTL is a potentially fatal disease second only to primary brain lymphoma; median survival is between 12 and 24 months [9]. It is due to failures in contralateral testis, CNS, and extranodal sites. Recently, an international phase II trial showed that the recommended first-line treatment for stage I or II PTL is orchiectomy followed by R-CHOP, IT-MTX, and RT to the contralateral testis [10]. In the same way, it has been said that multidisciplinary treatments should be given in early staged spermatic cord lymphoma, but actually was not. Reviewing reported cases of stage I spermatic cord lymphoma, the initial treatment was only orchiectomy with or without RT by the 1980s, and the ratio of combining chemotherapy or not has become half and half since the 1990s. No case except ours underwent IT-MTX. Median survival of stage I spermatic cord lymphoma treated only orchiectomy with or without RT was 18.2 months, while 5-year overall survival rates of early staged PTL treated multidisciplinarily were 85% in the recent trial [10]. It suggests that combined treatment with R-CHOP, IT-MTX, and RT to the contralateral testis will attribute a better outcome in early staged spermatic cord lymphoma.

On the other hand, the treatment of choice for stage III or IV disease in spermatic cord lymphoma still remains to be determined. For advanced PTL, the recommended first-line treatment is R-CHOP, however a median survival of less than six months is reported [9]. Despite a higher rate of relapse in the contralateral testis (up to 50% of patients), RT to the contralateral testis is recommended by some authors, while others propose RT only for symptomatic patients or in the case of bulky disease. IT-MTX to prevent CNS relapse, which occurs in approximately 50% of patients, is also considered in patients achieving complete remission.

## Figures and Tables

**Table 1:** Reported cases of spermatic cord lymphoma.

	Author	Age, *y*	Stage	Histology	Immuno-phenotype	Initial treatment	Survival, mo
1934	Fresnais	73	IE	LS	NA	O	DOD, 5
1936	Slotkin	36	IE	LS	NA	O	DOD, 1
1949	Hector	64	IE	RES	NA	O	DOD, 62
1954	Wetterwald	49	IIE	LB	NA	O	DOD, 1
1957	Pelot	21	IE	RS	NA	O, RT	DOD, 12
1970	Bologna	42	IE	LRS	NA	O, RT	DOD, 6
1970	Gotou	57	IE	RS	NA	O, RT	NA
1972	Iwata	30	IIE	LS	NA	O, CT	DOD, 3
1972	Katou	75	III	RS	NA	O, CT	DOD, 3
1980	Karapandov	57	IE	CB/CC	NA	O, RT	NA
1982	Guena	46	IE	CB/CC	NA	NA	NA
1982	Satou	49	IE	Burkitt	B	O, CT	NA
1986	Hautzer	54	IE	CBD	NA	O	DOD, 7
1986	Hanada	74	IE	DLC	NA	O	DOD, 5
1989	Zwanger-Mendelson	20	IV	Burkitt	B	O	DOD, 1
1989	Ooyama	66	IIE	DLC	NA	O, CT	NA
1990	D'Abrosca	89	IE	CBD	NA	NA	DOD, NA
1990	Nishimura	47	IIE	DLC	NA	O, CT	NA
1994	Möller	48	IV	CBD	B	NA	DOD, 8
1994	Kawanishi	66	IE	DMC	NA	O, CT, RT	NA
1994	Asanuma	78	IE	DLC	NA	CT	NA
1994	Oonishi	76	IE	DMC	NA	O	NA
1996	Lands	57	IE	DLC	B	O	DOD, 26
1997	Umehara	78	IE	DLC	NA	O	NA
1998	Ichiyanagi	77	IIE	DLC	B	O, CT	DOD, 10
2001	Okabe	76	IE	DMC	B	O	DOD, 40
2007	Kawano	57	IIE	DLBCL	B	O, CT	NA
2008	Nakano	54	IVA	DLBCL	B	O, CT	DOD, 5
2009	Natsuizaka	75	IE	DLBCL	B	O, CT	NA
2009	Almeida	71	IE	DLBCL	B	O, CT	NA
2009	Suzuki	74	IIIA	DLBCL	B	O, CT	DOD, 13
2009	Asano	71	IE	DLBCL	B	O, CT	NA
2011	Our case	62	IVB	DLBCL	B	O, CT, RT, IT	NA

O, orchiectomy; CT, chemotherapy; RT, radiotherapy; IT, intrathecal chemotherapy; CBD, centroblastic diffuse; CB/CC, centroblastic/centrocytic; LB, lymphoblastic; LS, lymphosarcoma; RS, reticulosarcoma; LRS, lymphoreticulosarcoma; RES, reticuloendotheliosarcoma; DLC, diffuse large cell; DMC, diffuse medium cell; DLBCL, diffuse large B-cell lymphoma; mo, months; NA, not available; DOD, died of disease.
